# A Full Ranking for Decision Making Units Using Ideal and Anti-Ideal Points in DEA

**DOI:** 10.1155/2014/282939

**Published:** 2014-07-24

**Authors:** A. Barzegarinegad, G. Jahanshahloo, M. Rostamy-Malkhalifeh

**Affiliations:** Department of Mathematics, Science and Research Branch, Islamic Azad University, Tehran, Iran

## Abstract

We propose a procedure for ranking decision making units in data envelopment analysis, based on ideal and anti-ideal points in the production possibility set. Moreover, a model has been introduced to compute the performance of a decision making unit for these two points through using common set of weights. One of the best privileges of this method is that we can make ranking for all decision making units by solving only three programs, and also solving these programs is not related to numbers of decision making units. One of the other advantages of this procedure is to rank all the extreme and nonextreme efficient decision making units. In other words, the suggested ranking method tends to seek a set of common weights for all units to make them fully ranked. Finally, it was applied for different sets holding real data, and then it can be compared with other procedures.

## 1. Introduction

Data envelopment analysis (DEA) is a nonparametric method to define the relative efficiency of a group of decision making units (DMUs) that use multiple inputs to produce multiple outputs. Methodology of DEA pioneered by Farrell [1] and later developed by Charnes et al. [2]. DEA computes the relative efficiencies of all DMUs by finding a set of the best weights for every DMU by maximizing its efficiency. On the other hand, flexibility in picking different inputs and outputs weights leads to coming up with DMUs as relative efficient which causes ranking disorders among DMUs. To reduce the flexibility in selecting inputs and outputs weights, researchers have already tried to modify the traditional DEA model and remove the weak points as well. Here are listed some of these offered methods. The assurance region model was firstly presented by Thompson et al. [3] and the common weights model by Roll et al. [4]. Roll and Golany [5] offered an alternative method in which they normalized all inputs and outputs at the beginning, in a way that the magnitude of parameters would not influence the model and then through imposing restrictions on the weights of the model, they could achieve common weights. Mavi et al. [6] presented a common set of weights using ideal point method. Hosseinzadeh Lotfi et al. [7] and Jahanshahloo et al. [8] proposed two different models in DEA by using common weights. They suggested that instead of solving *n* linear programming models, we can reach the efficiency of DMUs through solving only one nonlinear programming model. Through utilizing multiple objective programming (MOP) and common set of weights (CSW), Hosseinzadeh Lotfi et al. [7] introduced a model to compute the efficiency of DMUs.

When DEA models are applied to calculate the performance of DMUs, usually several DMUs yield with the same efficiencies, that are all equal to one. Therefore, it is necessary to suggest a model to differentiate between these units. Otherwise, we are not able to rank them accordingly. Numerous models have been proposed to reduce the number of efficient units so far: Andersen and Petersen (AP) [9] and Mehrabian, Alirezaee, and Jahanshahloo (MAJ) [10] can be considered as two of the most popular of these methods; however, sometimes they fail in ranking. So we intend to compare the proposed procedure with the two aforementioned methods by some examples in this paper. Additionally, some papers based on cross-efficiency have been prepared such as Sexton et al. [11], Wu et al. [12], Jahanshahloo et al. [13, 14], and Wang et al. [15].

One of the most important and practical procedures in ranking is benchmarking methods, which are suggested by Torgesen et al. [16], Sueyoshi [17], Lu and Lo [18], and Chen and Deng [19]. Wang et al. [20] presented two nonlinear programming models for full ranking which have high complexity for computations. You can see [21] for further study on ranking methods. To overcome the problems in the complete ranking of units, we propose a mixed integer programming which is capable of ranking every (extreme and nonextreme) efficient DMU, although sometimes other methods fail. The main purpose in this paper is introducing a model to evaluate the DEA efficiency of DMUs. We tend to suggest ideal and anti-ideal points in the model; then through using CSW and MOP a comprehensive evaluation of DMUs can be proposed. In addition, we prove that our model is feasible. The rest of this paper is organized as follows.  Section 2 briefly introduces the approach of finding a CSW by MOP concepts. In Section 3, a procedure would be proposed to rank DMUs.  Section 4 compares the proposed method with the other models using three numerical examples. The paper is concluded in the final section.

## 2. Common Set of Weights Model

Assume that there is a set of *n* DMUs. Each DMU_*j*_ (*j* = 1,…, *n*) consumes the amounts *X*_*j*_ = {*x*_*ij*_} of *m* different of inputs (*i* = 1,…, *m*) and produces the amounts *Y*_*j*_ = {*y*_*rj*_} of *r* outputs (*r* = 1,…, *s*). Charnes et al. [2] presented following well-known CCR model which measures the relative efficiencies of DMUs:

(1) max⁡ θo=∑r=1suryro∑i=1mvixios.t.     θj=∑r=1suryrj∑i=1mvixij≤1, j=1,…,nur≥ɛ, vi≥ɛ, r=1,…,s, i=1,…,m,

where DMU_*o*_ represents the DMU under evaluation; *u*_*r*_  (*r* = 1,…, *s*) and *v*_*i*_  (*i* = 1,…, *m*) are the weights assigned to the outputs and inputs and *ɛ* presents a non-Archimedean infinitesimal. If there is a set of positive weights that makes *θ*_o_* = 1, then DMU_*o*_ is called relative efficient and otherwise it is called relative inefficient. The linear programming equivalent of model (1) is

(2) max⁡ ∑r=1suryros.t.    ∑r=1suryrj−∑i=1mvixij≤0, j=1,…,n,∑i=1mvixio=1,ur≥ɛ, vi≥ɛ, r=1,…,s, i=1,…,m.

This problem has a dual which is given by

(3) min⁡ θo−ɛ(∑i=1msi−+∑r=1ssr+)s.t.   ∑j=1nλjxij+si−=θoxio, i=1,…,m,∑λjyrj−sr+=yro, r=1,…,s,λj,si−,sr+≥0, j=1,…,n,i=1,…,m, r=1,…,s.

The constraint space of (3) defines the production possibility set (PPS) *T*_*c*_. That is,

(4) Tc={(x,y) ∣ x≥∑j=1nλjxj,  y≤∑j=1nλjyj,  λj≥0, j=1,…,n}.

It should be noted that DMU_*o*_ is extreme efficient if and only if the model (3) has a unique optimal solution as follows:

(5) θo∗=1,λo∗=1,λj∗=0, j=1,…,n,  j≠o,s−∗=0,  s+∗=0.

Extra flexibility to choose weights mostly brings several DMUs with relative efficient DMUs. However, to remove this problem, many attempts have been explored further restricting weights in DEA. One of the most important ones is the common weights method in DEA, which at first initiated by Cook et al. [22]. The other method was proposed by Roll et al. [4] in DEA, where all DMUs can be evaluated by only one common weight. While it is almost tough, it can suggest more precise ranking; therefore each introduced efficient DMU of this method would be efficient DMU in primary DEA models. Hosseinzadeh Lotfi et al. [7] suggested a model to compute the efficiency of DMUs, in which they were only solved by one nonlinear programming model instead of *n* linear programming models. The following multiobjective fractional programming (MOFP) can be used to maximize the efficiency score of all DMUs together [7]:

(6) max⁡ {∑r=1suryr1∑i=1mvixi1,∑r=1suryr2∑i=1mvixi2,…,∑r=1suryrn∑i=1mvixin}s.t.    ∑r=1suryrj∑i=1mvixij≤1, j=1,…,n,ur≥ɛ, vi≥ɛ, r=1,…,s, i=1,…,m.

Several methods have been proposed to solve the aforementioned MOFP problem. One of them is goal programming (GP). Based on the GP method, model (6) can be transformed to the following model for attaining a set of common weights [23]:

(7) min⁡ ∑j=1n(nj+pj)s.t.   ∑r=1suryrj∑i=1mvixij+nj−pj=Aj, j=1,…,n,∑r=1suryrj∑i=1mvixij≤1, j=1,…,n,ur≥ɛ, vi≥ɛ, nj≥0, pj≥0, r=1,…,s, i=1,…,m, j=1,…,n.

Here *A*_*j*_ is the goal of the *j*th objective function and *n*_*j*_, *p*_*j*_ represent the positive deviation and negative deviation of the *j*th goal, respectively. On the other hand, while in the conventional DEA models, every individual DMU tends to maximize its efficiency, so the amounts of *A*_*j*_  (*j* = 1,…, *n*) in model (7) would be one.

## 3. The Proposed Ranking Method

On the first step we are going to introduce ideal and anti-ideal points.

**Definition 1 .**

An ideal point is a point that can consume the least inputs to produce the most outputs.

**Definition 2 .**

An anti-ideal point is a point that uses the most inputs only to generate the least outputs.

Due to mentioned definitions we can show the inputs and outputs of ideal point with *x*_*i*_^min⁡^  (*i* = 1,…, *m*) and *y*_*r*_^max⁡^  (*r* = 1,…, *s*), respectively. Also, we denote by *x*_*i*_^max⁡^  (*i* = 1,…, *m*) and *y*_*r*_^min⁡^  (*r* = 1,…, *s*) the inputs and outputs of anti-ideal point, respectively. These are determined as follows:

(8) ximin⁡=min⁡j{xij},  ximax⁡=max⁡j{xij}, i=1,…,m, j=1,…,n,yrmin⁡=min⁡j{yrj},  yrmax⁡=max⁡j{yrj}, r=1,…,s, j=1,…,n.

According to efficiency concept, the efficiency of ideal point can be defined as

(9) $$\begin{array}{c} \theta_{I}=\frac{\sum_{r=1}^{s}u_{r}y_{r}^{\max}}{\sum_{i=1}^{m}v_{i}x_{i}^{\min}}, \end{array}$$

where *u*_*r*_,  *v*_*i*_ are the weights assigned to the *r*th output and the *i*th input, respectively. Suppose that *θ*_I_* is the ideal point efficiency, which results from the following LP model:

(10) max⁡ θI=∑r=1suryrmax⁡s.t.   ∑r=1suryrj−∑i=1mvixij≤0, j=1,…,n,∑i=1mviximin⁡=1,ur≥ɛ, vi≥ɛ, r=1…,s, i=1,…,m.

As such, the efficiency score of anti-ideal point can be specified as

(11) $$\begin{array}{c} \theta_{A}=\frac{\sum_{r=1}^{s}u_{r}y_{r}^{\min}}{\sum_{i=1}^{m}v_{i}x_{i}^{\max}}. \end{array}$$

If we consider *θ*_A_* as the efficiency of the anti-ideal point, then it can be solved by the model below:

(12) max⁡ θA=∑r=1suryrmin⁡s.t.    ∑r=1suryrj−∑i=1mvixij≤0, j=1,…,n,∑i=1mviximax⁡=1,ur≥ɛ, vi≥ɛ, r=1…,s, i=1,…,m.

Here we assume that *θ*_I_* is a goal for all DMUs, in such a way every single DMU tends to get its efficiency approach to it and consequently each DMU inclines toward getting its efficiency farther from *θ*_A_*. Then in accordance with this idea and goal programming,we obtain the following model:

(13) min⁡ ∑j=1n(nj−pj)s.t.   ∑r=1suryrj+nj∑i=1mvixij=θI∗, j=1,…,n,∑r=1suryrj−pj∑i=1mvixij=θA∗, j=1,…,n,∑r=1suryrj∑i=1mvixij≤1, j=1,…,n,ur≥ɛ, vi≥ɛ, nj≥0, pj≥0,r=1,…,s, i=1,…,m, j=1,…,n,

where *n*_*j*_ and *p*_*j*_ are deviation variables for the DMU_*j*_. Although model (13) is able to reduce the efficient units in DEA, it is still possible to evaluate more than one DMU as an efficient unit in DEA; therefore it cannot suggest a comprehensive ranking for *n* DMUs. To overcome this problem, the following model is offered:

(14) min⁡ ∑j=1n(nj−pj)s.t.   ∑r=1suryrj+nj∑i=1mvixij=θI∗, j=1,…,n,∑r=1suryrj−pj∑i=1mvixij=θA∗, j=1,…,n,∑r=1suryrj∑i=1mvixij≤1, j=1,…,n,∑r=1suryrj+tj∑i=1mvixij=1, j=1,…,n,ɛdj≤tj≤Mdj, j=1,…,n,∑j=1ndj=n−1, dj∈{0,1}, j=1,…,n,ur≥ɛ, vi≥ɛ, nj≥0, pj≥0,r=1,…,s, i=1,…,m, j=1,…,n,

where *M* is a giant number. In model (14) each DMU by minimizing its efficiency from ideal point efficiency and maximizing from anti-ideal point efficiency tends to attain a set of inputs and outputs weights.

**Definition 3 .**

If (u*, v*) is an optimal solution of model (2), then

(15) $$\begin{array}{c} H=\left\{\left(x,y\right)\;|\;u^{t*}y-v^{t*}x=0\right\} \end{array}$$

is a supporting hyperplane of the PPS.

**Theorem 4 .**

Model (14) is feasible and bounded.

**Proof**

For evaluating DMUs, we can use model (2). We know that there exists at least one extreme efficient unit by using model (2). Without loss of generality, suppose that the DMU_1_ is extreme efficient. On the other hand, there exist an infinite number of supporting hyperplanes passing through any extreme efficient DMU [24]. We will show that there exists a supporting hyperplane that only DMU_1_ lies on its intersection and *T*_*c*_. Since DMU_1_ is extreme efficient, hence model (3) has a unique optimal solution

(16) θ1∗=1,λ1∗=1,  λj∗=0, j=2,…,n,s−∗=0,  s+∗=0.

After including slack variables *t*_*j*_  (*j* = 1,…, *n*) in the first *n* constraints of model (2), according to strong complementary slackness conditions (SCSC) [25], there exist a pair of an optimal solution (u*, v*, t*) of model (2) and an optimal solution (θ*, λ*, *s* ^−∗^, *s* ^+∗^) of model (3), such that

(17) λj∗tj∗=0,  λj∗+tj∗>0, j=1,…,n,ur∗sr+∗=0,  ur∗+sr+∗>0, r=1,…,s,vi∗si−∗=0,  vi∗+si−∗>0, i=1,…,m,

where *t*_j_* = −∑_*r*=1_^*s*^ *u*_r_*y_*rj*_ + ∑_*i*=1_^*m*^ *v*_i_*x_*ij*_. Therefore, since (16) is the unique optimal solution model (3); then by (17) we have

(18) $$\begin{array}{c} t_{1}^{*}=0,\;\;t_{j}^{*}>0,\;j=2,\ldots ,n. \end{array}$$

Equation (18) implies that

(19) ∑r=1sur∗yr1−∑i=1mvi∗xi1=0,  ∑r=1sur∗yrj−∑i=1mvi∗xij<0,j=2,…,n.

That is,

(20) $$\begin{array}{c} \frac{\sum_{r=1}^{s}u_{r}^{*}y_{r1}}{\sum_{i=1}^{m}v_{i}^{*}x_{i1}}=1,\;\;\frac{\sum_{r=1}^{s}u_{r}^{*}y_{rj}}{\sum_{i=1}^{m}v_{i}^{*}x_{ij}}<1,\;j=2,\ldots ,n. \end{array}$$

Hence, only DMU_1_ lies on the supporting hyperplane *H* = {(*x*, *y*)∣*u* ^∗*t*^ *y* − *v* ^∗*t*^ *x* = 0}. It is evident that (21) is a feasible solution for model (14):

(21) ur=ur∗, vi=vi∗, r=1,…,s, i=1,…,m,d1=0,  dj=1, j=2,…,n,t1=0,  tj>0, j=2,…,n,nj=θI∗∑i=1mvixij−∑r=1suryrj, j=1,…,n,pj=∑r=1suryrj−θA∗∑i=1mvixij, j=1,…,n.

On the other hand, since *n*_*j*_ ≥ 0 and 0 ≤ *θ*_A_* ≤ 1, obviously model (14) is bounded. This completes the proof.

Let *u*_r_*  (*r* = 1,…, *s*) and *v*_i_*  (*i* = 1,…, *m*) be the optimal weights in model (14). Then

(22) $$\begin{array}{c} \theta_{j}^{*}=\frac{\sum_{r=1}^{s}u_{r}^{*}y_{rj}}{\sum_{i=1}^{m}v_{i}^{*}x_{ij}},\;j=1,\ldots ,n, \end{array}$$

is referred to as the efficiency of DMU_*j*_. Model (14) introduces a complete ranking for *n* DMUs and this will be tested and illustrated in the next section through some examples.

## 4. Numerical Examples

In this section, we provide three numerical examples that all involve a significant number of DEA efficient units. Then we compare them to other methods to show the potential usage of the proposed ranking methodology in the complete ranking of DMUs.

**Example 1 .**

Consider the 12 flexible manufacturing systems (FMSs) given in Table 1 with two inputs and four outputs. These data are taken from Shang and Sueyoshi [26]. We solve proposed model for all FMSs and compare results with the Wang et al. [20] and Kao and Hung models [27]. Seeing the results in Table 1, the researcher can realize seven FMSs as CCR efficient FMSs. Therefore, it is impossible to have complete ranking for all FMSs. In order to solve this problem, Kao and Hung [27] suggested to use three common weights of DEA models. The gained results are shown in columns 2, 3, and 4 in Table 2. It is easily understood that in the first model of Kao and Hung [27], four FMSs and other models just generate two FMSs as efficient FMSs. Moreover, these three models illustrate three different ranking, which is a demerit of the proposed models by Kao and Hung [27].

However, the main deficiency in Kao and Hung [27] is that the proposed models related to *p* as a parameter which is calculated in Example 1 as *P* = 1, *P* = 2, and *P* = *∞*. Wang et al. [20] suggested two models utilizing common weights which to some extent eliminate deficiencies of the Kao and Hung models. As it can be seen in Table 2, Wang et al. [20] evaluate FMS_5_ as the efficient FMS. The resulted efficiencies and rankings by Wang models are inserted in the fifth and sixth columns of Table 2. Although the two proposed models by Wang et al. [20] have the same ranking, this is not right always. On the other hand, the introduced models are nonlinear ones. Although their method is an interesting approach as a theoretical idea, it could not be efficient from computational point of view. The last column in Table 2 illuminates the results of efficiency and ranking obtained by the proposed model of the paper. It is clear from Table 2 that the proposed model with ideal and anti-ideal points considers FMS_5_ as the efficient FMS, and all the other 11 FMSs as nonefficient. As you may notice from Table 2, FMS_9_ has the worst performance by Wang et al. and suggested models.

**Example 2 .**

Consider the example investigated by Jahanshahloo et al. [28] where all DMUs have two inputs and two outputs. The data are reproduced in Table 3, together with the CCR efficiencies of seven DMUs. The example is solved by two methods: AP and MAJ which are the most popular methods. In accordance with Table 3, the results of ranking by proposed method are almost the same with AP and MAJ ranking methods. On the other hand, we can achieve correlation analysis of the efficiencies gained by imposing these three models.  Table 4 states results of analysis. This research has been significant at the level of 0.01. As you can see in Table 4, there is a high correlation between the proposed model and AP and MAJ models. It is worth notifying that if AP and MAJ models are infeasible or unable to present a complete ranking, these three models lack this correlation and ranking declines to its lowest level of correlation analysis. So, please notice the next example.

**Example 3 .**

**Figure 1:**
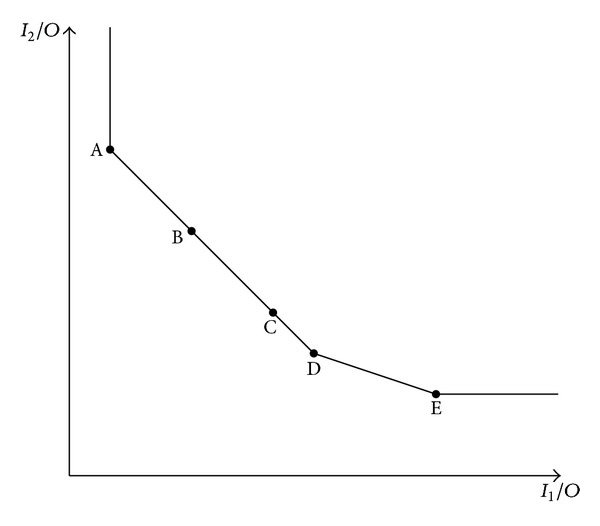
Farrell frontier for five DMUs.

Consider the problem of measuring the performances of five DMUs, where each DMU has two inputs and one output. The data set is shown in Table 5. Here all outputs have been normalized to one for convenience. The Farrell frontier for these DMUs is shown in Figure 1. As can be seen in Figure 1, DMU_A_, DMU_D_, and DMU_E_ are extreme efficient DMUs and DMU_B_ and DMU_C_ are nonextreme efficient DMUs. The results of this example by using our proposed method, *θ*_AP_* and *θ*_MAJ_*, are documented in Table 5. As you can see in Table 5, AP and MAJ models could not rank DMU_*B*_ and DMU_*C*_; however the proposed model here is able to rank both extreme and nonextreme efficient DMUs. It shows the first privilege of the new ranking model over AP and MAJ models. According to the suggested model, performance of the five DMUs is ranked as follows:

(23) $$\begin{array}{c} {\text{DMU}}_{\text{A}}≻{\text{DMU}}_{\text{B}}≻{\text{DMU}}_{\text{C}}≻{\text{DMU}}_{\text{D}}≻{\text{DMU}}_{\text{E}}, \end{array}$$

where “≻” denotes superior to. The second advantage of the proposed method is that to obtain a complete ranking for all DMUs; the researcher just needs to solve three programs, though the other models lack this merit. The third privilege is its feasibility; however, in some cases the AP and MAJ models are infeasible. Utilizing correlation analysis at the 0.01 level, then correlation between the suggested method and AP, MAJ is recorded as 0.478 and 0.185, respectively. This poor correlation is for this fact that AP and MAJ are not able to present a comprehensive ranking.

## 5. Conclusion 

In the current paper, we have developed a new mixed integer programming based on ideal and anti-ideal points. In this procedure, firstly we must compute ideal and anti-ideal points to rank all DMUs. Then their efficiency scores could be obtained. Through using the proposed model, all DMUs can be ranked, whereas most of ranking methods cannot do it. One of the prominent features of this model compared to the others is that it is always feasible. On the other hand, traditional DEA models cannot define a DMU with the best performance. However, it can be easily conducted by the proposed model here. The other advantage of this new model is that we are able to rank all the extreme and nonextreme efficient units by solving only three programs. Three numerical examples have been tested and examined by applying the suggested ranking method. The proposed model complies with crisp data. It can be examined further in the future researches in accordance with interval or fuzzy data.

## Figures and Tables

**Table 1:** Data and CCR efficiency for Example 1.

FMS	Inputs		Outputs				CCR efficiency
	*X*_1_	*X*_2_	*Y*_1_	*Y*_2_	*Y*_3_	*Y*_4_	
1	17.02	5	42	45.3	14.2	30.1	1
2	16.46	4.5	39	40.1	13	29.8	1
3	11.76	6	26	39.6	13.8	24.5	0.9682
4	10.52	4	22	36	11.3	25	1
5	9.50	3.8	21	34.2	12	20.4	1
6	4.79	5.4	10	20.1	5	16.5	1
7	6.21	6.2	14	26.5	7	19.7	1
8	11.12	6	25	35.9	9	24.7	0.9614
9	3.67	8	4	17.4	0.1	18.1	1
10	8.93	7	16	34.3	6.5	20.6	0.9536
11	17.74	7.1	43	45.6	14	31.1	0.9831
12	14.85	6.2	27	38.7	13.8	25.4	0.8012

**Table 2:** The results of Example 1.

FMS	Kao and Hung models [27]			Wang et al. models [20]		Proposed method
	Common model (1)	Common model (2)	Common model (3)	Model (5)	Model (6)	
1	1.0000 (1)	0.9654 (4)	0.9111 (6)	1.0101 (5)	1.0051 (5)	0.8680 (7)
2	0.9766 (5)	0.9616 (6)	0.9026 (7)	0.9867 (7)	0.9818 (7)	0.8370 (9)
3	0.9488 (9)	0.9132 (9)	0.9021 (8)	1.0185 (4)	1.0134 (4)	0.9256 (5)
4	1.0000 (1)	1.0000 (1)	1.0000 (1)	1.0702 (2)	1.0649 (2)	0.9783 (3)
5	1.0000 (1)	0.9641 (5)	0.9663 (4)	1.0893 (1)	1.0893 (1)	1.0000 (1)
6	0.9624 (6)	0.9866 (3)	0.9872 (3)	1.0058 (6)	1.0008 (6)	0.9481 (4)
7	1.0000 (1)	1.0000 (1)	1.0000 (1)	1.0481 (3)	1.0429 (3)	0.9853 (2)
8	0.9614 (7)	0.9423 (7)	0.9203 (5)	0.9752 (8)	0.9704 (8)	0.8832 (6)
9	0.7528 (12)	0.8462 (10)	0.8760 (9)	0.7190 (12)	0.7155 (12)	0.7462 (12)
10	0.8334 (10)	0.8041 (11)	0.8295 (11)	0.8521 (10)	0.8478 (10)	0.8374 (8)
11	0.9507 (8)	0.9160 (8)	0.8591 (10)	0.9528 (9)	0.9481 (9)	0.8173 (10)
12	0.7943 (11)	0.7750 (12)	0.7602 (12)	0.8501 (11)	0.8460 (11)	0.7618 (11)

**Table 3:** Inputs and Outputs and ranking by AP, MAJ, and new proposed ranking models.

DMU	Input 1	Input 2	Output 1	Output 2	CCR efficiency	*θ*_AP_*	*θ*_MAJ_*	*θ*_New _*
A	2	3	4	5	0.9254 (4)	0.9286 (4)	0.9524 (4)	0.8667 (3)
B	3	4	5	6	0.7602 (5)	0.7619 (5)	0.7619 (5)	0.7173 (5)
C	2	2	4	4	0.9900 (3)	1.0000 (3)	1.0000 (3)	0.8571 (4)
D	2	3	5	4	1.0000 (1)	1.2500 (2)	1.1667 (2)	0.9333 (2)
E	3	4	4	5	0.6174 (7)	0.6100 (7)	0.6190 (7)	0.5909 (7)
F	2	2	4	6	1.0000 (1)	1.5000 (1)	1.2857 (1)	1.0000 (1)
G	3	4	5	4	0.7093 (6)	0.7143 (6)	0.7143 (6)	0.6364 (6)

**Table 4:** Result of correlation analysis.

	Proposed method	AP method	MAJ method
Proposed method	1	0.945	0.982
AP method	/	1	0.986
MAJ method	/	/	1

Correlation is significant at the 0.01 level (two-tailed).

**Table 5:** Data for Example 3 and their ranking and efficiencies by AP, MAJ, and new proposed ranking models.

DMU	Input 1	Input 2	Output 1	*θ*_AP_*	*θ*_MAJ_*	*θ*_New _*
A	1	8	1	3.0000 (1)	1.2222 (2)	1.0000 (1)
B	3	6	1	1.0000 (4)	1.0000 (4)	0.9048 (2)
C	5	4	1	1.0000 (4)	1.0000 (4)	0.8261 (3)
D	6	3	1	1.0833 (3)	1.0400 (3)	0.7917 (4)
E	9	2	1	1.5000 (2)	1.1250 (1)	0.6129 (5)
